# Waist-hip ratio is superior to BMI in predicting liver-related outcomes and synergizes with harmful alcohol use

**DOI:** 10.1038/s43856-023-00353-2

**Published:** 2023-09-06

**Authors:** Fredrik Åberg, Martti Färkkilä, Veikko Salomaa, Antti Jula, Satu Männistö, Markus Perola, Annamari Lundqvist, Ville Männistö

**Affiliations:** 1 Transplantation and Liver Surgery, Helsinki University Hospital and University of Helsinki, Helsinki, Finland; 2 Abdominal Center, Helsinki University and Helsinki University Hospital, Helsinki, Finland; 3 Finnish Institute for Health and Welfare, Helsinki, Finland; 4 Departments of Medicine, University of Eastern Finland and Kuopio University Hospital, Kuopio, Finland

**Keywords:** Non-alcoholic fatty liver disease, Alcoholic liver disease

## Abstract

**Background:**

Obesity is associated with liver disease, but the best obesity-related predictor remains undefined. Controversy exists regarding possible synergism between obesity and alcohol use for liver-related outcomes (LRO). We assessed the predictive performance for LROs, and synergism with alcohol use, of abdominal obesity (waist-hip ratio, WHR), and compared it to overall obesity (body mass index, BMI).

**Methods:**

Forty-thousand nine-hundred twenty-two adults attending the Finnish health-examination surveys, FINRISK 1992–2012 and Health 2000 studies, were followed through linkage with electronic healthcare registries for LROs (hospitalizations, cancers, and deaths). Predictive performance of obesity measures (WHR, waist circumference [WC], and BMI) were assessed by Fine-Gray models and time-dependent area-under-the-curve (AUC).

**Results:**

There are 355 LROs during a median follow-up of 12.9 years (509047.8 person-years). WHR and WC emerge as more powerful predictors of LROs than BMI. WHR shows significantly better 10-year AUC values for LROs (0.714, 95% CI 0.685–0.743) than WC (0.648, 95% CI 0.617–0.679) or BMI (0.550, 95% CI 0.514–0.585) both overall and separately among men and women. WHR is predictive also in BMI strata. Absolute 10-year risks of LROs are more dependent on WHR than BMI. Moreover, WHR shows a significant supra-additive interaction effect with harmful alcohol use for liver-related outcomes (excess 10-year cumulative incidence of 2.8% from the interaction), which is not seen between BMI and harmful alcohol use.

**Conclusions:**

WHR is a better predictor than BMI or WC for LROs, and WHR better reflects the synergism with harmful alcohol use. WHR should be included in clinical assessment when evaluating obesity-related risks for liver outcomes.

## Plain language summary

Obesity has been linked to liver disease, but the most accurate measure for predicting obesity-related liver disease outcomes remains uncertain. In this study, we analyzed data from over 40,000 adults to compare the extent to which different measures of obesity can predict liver-related outcomes, such as severe liver disease, liver failure, or death from liver disease. The measures of obesity were the ratio of waist circumference to hip circumference (waist-hip ratio, WHR), waist circumference (WC), and body mass index (BMI). Our findings reveal that WHR and WC are stronger predictors of these outcomes than BMI. In particular, WHR demonstrated superior predictive ability and this predictive ability was influenced by harmful alcohol use. This study suggests that WHR may be a relatively simple but useful measure for clinicians to use when predicting obesity-related risks for liver health.

## Abstract (web summary)

Åberg et al. assess the predictive performance of obesity measures, including waist-hip ratio, waist circumference, and BMI, for liver-related outcomes in participants of Finnish health surveys. They find that the waist-hip ratio is a better predictor for liver-related outcomes than other measures and synergizes with harmful alcohol use.

## Introduction

There is a dose-dependent relationship between the level of obesity and the risk for liver disease^1^. However, the anthropometric measure that best predicts future liver disease remains unclear. While most studies have analyzed body mass index (BMI), it is now increasingly appreciated that the waist-hip ratio (WHR) better reflects fat distribution and abdominal obesity and as such seems to reflect metabolic health better than the BMI^2^. Hip circumference mirrors lower-body subcutaneous fat mass, which is not as metabolically harmful as visceral fat mass in the abdominal region, and may even have protective effects^2,3^. Waist and hip circumference have independent and opposite associations with incident liver disease, and these effects seem to be largely captured in the WHR^4^. The WHR has been shown to be the obesity indicator with the highest predictive capacity for non-alcoholic fatty liver disease (NAFLD)^5^. In addition, the WHR has been shown to predict severe liver disease and liver-related outcomes better than BMI, but population studies are still scarce^6–8^, and previous studies have not used competing-risk analysis.

In addition to obesity, there are also dose-dependent relationships between alcohol consumption and the risk for liver disease^9^. Moreover, many studies have pointed to supra-additive interaction effects between alcohol and obesity for markers of liver disease and for liver-related outcomes^10,11^. Supra-additive interaction basically means that the combined risk effect of two concurrent exposures (for instance, harmful alcohol use and obesity) on the risk for liver disease is greater than the sum of their individual risk effects. Nonetheless, controversy remains regarding whether such interaction effects truly exist or not^11–13^. A recent systematic review failed to find supra-additive interaction effects between alcohol and BMI for liver disease^12^. However, there are several methodological concerns with the previous studies, including the reliance on self-reported data, often small sample sizes, and a lack of competing risk analyses and absolute risk estimates.

We assessed the predictive performance for liver-related outcomes of WHR, and compared it to that of waist circumference (WC) and BMI. We further compared the interaction effects between harmful alcohol use and WHR or BMI. Finally, we demonstrate how WHR affects the absolute risk of liver-related outcomes substantially more than the BMI when other parameters are kept constant. WHR emerged as a better predictor than BMI or WC for liver-related outcomes, and WHR better reflects the synergism with harmful alcohol use.

## Methods

Data were sourced from the Finnish health-examination studies, FINRISK 1992–2012, and Health 2000 Survey. FINRISK studies are national population surveys carried out in Finland every 5 years by the Finnish Institute for Health and Welfare, using random representative population samples^14^. The Health 2000 Survey was similarly coordinated by the Finnish Institute for Health and Welfare, and originally comprised 8028 adults aged 30 years and above; the participation rate in the full examinations was 80%^15^.

Data collection, sample formation, and linkage with electronic healthcare registers for liver-related outcomes have been previously described^4,14,15^. Briefly, weight, height, waist, and hip circumference were all measured at baseline, i.e., at the time of the health-examination. Alcohol use was assessed by standard questionnaires. Individuals were linked with the Care Register for Health Care (HILMO) for hospitalizations, with the Finnish Cancer Registry for malignancies, and with the Statistics Finland register for vital status and cause of death until December 2015. A liver-related outcome was defined by ICD-9 and ICD-10 codes reflecting severe liver disease (requiring hospital admission or causing liver cancer, or liver-related death) in line with a recent consensus paper^16^; the specific ICD codes used are presented in Supplementary Note 1.

All participants provided signed informed consent, and the studies were approved by the Coordinating Ethical Committee of the Helsinki and Uusimaa Hospital District. Previously, the studies were also approved by the institutional review board of the National Public Health Institute in Helsinki, Finland. The FINRISK 1992–2012 Health 2000 sample collections were transferred to THL Biobank in 2015 according to the Finnish Biobank Act.

### Statistical methods

For comparing baseline characteristics between sex groups, we used Chi-Square or Mann-Whitney tests as appropriate. Correlations were calculated using the Spearman method. Associations between WHR, WC, or BMI with liver-related outcomes were assessed by Fine-Gray regression analyses, and non-linear associations by restricted cubic splines. We evaluated the discrimination performance of WHR, WC, and BMI for liver-related outcomes in terms of time-dependent area-under-the-curve (AUC) values at 10 years of follow-up based on Fine-Gray competing-risk regression models, where death without liver disease was considered a competing-risk event. Models were compared statistically by delta-AUCs and the Wald test using the methodology described by Blanche et al.^17^. We also evaluated the discrimination performance of WHR and WC in BMI strata to see if the performance of these obesity measures depended on the BMI.

To illustrate how the absolute risk of liver-related outcomes vary by WHR and BMI when age, sex, and alcohol use are kept constant, we constructed a Fine-Gray competing-risk model, separately for men and women, with age, alcohol use, WHR, and BMI as independent variables. Based on this model, we predicted the 10-year absolute risk for liver-related outcomes for a set of pre-specified parameters (BMI 25 or 35 kg/m^2^, and WHR 1.2 or 0.9 for men or 1.0 or 0.7 for women). We repeated this procedure in the subgroup of individuals with a high risk of having advanced liver fibrosis at baseline. Here, fibrosis risk was determined using the dynamic aspartate-to-alanine aminotransferase ratio (dAAR score), which is an externally validated score that is associated with both advanced fibrosis/cirrhosis and liver-related outcomes^18^. High risk was defined as a dAAR score above 2.63, in line with the original publication^18^.

To analyze whether supra-additive interaction effects exist between WHR or BMI and harmful alcohol use for liver-related outcomes, we applied the methodology recently described by Innes et al.^13^. First, we stratified WHR, BMI, and alcohol use into three levels. WHR was stratified into sex-specific tertiles (cutoffs for men: 0.93 and 0.99; cutoffs for women: 0.80 and 0.86) because there are no firmly established WHR cutoffs. BMI was stratified into normal weight (BMI 20–24.9 kg/m^2^), overweight (BMI 25–29.9 kg/m^2^), and obese (BMI ≥30 kg/m^2^) categories, and alcohol use according to the UK guidelines for safe, hazardous, and harmful drinking by the following cutoffs (men: 176 and 392 grams of ethanol/week; women: 120 and 280 grams/week)^19^. The reference group was those with safe alcohol use and no overweight or obesity (BMI 20–24.9 kg/m^2^ or lowest WHR tertile). Then, using the nonparametric cumulative incidence function, we calculated the 10-year excess cumulative incidence of liver-related outcomes by subtracting the cumulative incidence observed in a specific group from the cumulative incidence in the reference group. We repeated this procedure to calculate relative risks by Fine-Gray regression models adjusted for age, sex, education level, and employment and marital status. A two-tailed *P*-value < 0.05 was considered statistically significant. Data were analyzed with R software version 3.6.1.

### Reporting summary

Further information on research design is available in the Nature Portfolio Reporting Summary linked to this article.

## Results

### Study population

The initial combined sample from all surveys comprised 43,105 individuals. After excluding individuals with missing registry linkage (*n* = 1457), baseline liver disease (ICD-10 codes K70-K77 or C22; *n* = 299), chronic viral hepatitis (*n* = 89), or missing anthropometric measurements (*n* = 338), the final study cohort comprised 40,922 individuals.

Baseline characteristics of the 40,922 study participants are shown in Table 1. Mean age was 50 years, 47% were men, and the mean BMI was 26.8 kg/m^2^. Mean WHR was 0.96 among men and 0.84 among women. Mean WC was 96.3 cm among men 84.9 cm among women. Mean alcohol use was 75 grams of ethanol per week (around 7.5 standard units per week), 115 g/week for men and 39 g/week for women. Of the participants, 86% were in the lowest alcohol use strata (low alcohol use), 10% in the middle, and 4% in the highest strata (harmful alcohol use). For WHR, the distribution from the lowest to highest strata were 31%, 38%, and 31% respectively, and for BMI, 35%, 40%, and 21%, respectively; another 4% had a BMI < 20 kg/m^2^.

**Table 1:** Baseline demographics.

	Overall	Men	Women	*P*
Participants	40,922	19,399	21,523	
Age, years	49.6 (13.8)	49.9 (13.6)	49.3 (14.0)	<0.001
Education				0.001
Low	13,403 (33.2)	6179 (32.3)	7224 (34.0)	
Average	13,243 (32.8)	6419 (33.6)	6824 (32.1)	
High	13,701 (34.0)	6508 (34.1)	7193 (33.9)	
Marital status				<0.001
Married/partnership	29,654 (72.6)	14,737 (76.1)	14,917 (69.5)	
Single	5416 (13.3)	2835 (14.6)	2581 (12.0)	
Widow, separated, divorced	5764 (14.1)	1784 (9.2)	3980 (18.5)	
Employment status				<0.001
Part- or full-time employed	24,788 (61.4)	12,051 (63.1)	12,737 (59.8)	
Other^a^	5083 (12.6)	1801 (9.4)	3282 (15.4)	
Retired	10,526 (26.1)	5261 (27.5)	5265 (24.7)	
Diabetes	3097 (7.6)	1555 (8.0)	1542 (7.2)	0.001
Waist circumference, cm	90.3 (13.8)	96.3 (11.8)	84.9 (13.3)	<0.001
Hip circumference, cm	100.9 (9.1)	100.4 (7.5)	101.4 (10.3)	<0.001
Waist-hip ratio	0.89 (0.09)	0.96 (0.07)	0.84 (0.07)	<0.001
Waist-hip ratio category				<0.001
Lowest	12,703 (31.0)	6276 (32.4)	6427 (29.9)	
Middle	15,368 (37.6)	7290 (37.6)	8078 (37.5)	
Highest	12,851 (31.4)	5833 (30.1)	7018 (32.6)	
Body mass index, kg/m^2^	26.8 (4.7)	27.1 (4.1)	26.5 (5.1)	<0.001
Body mass index category, kg/m^2^				<0.001
<20	1434 (3.5)	288 (1.5)	1146 (5.3)	
20–24.9	14,353 (35.2)	5813 (30.1)	8540 (39.8)	
25–29.9	16,297 (40.0)	9172 (47.5)	7125 (33.2)	
≥30	8680 (21.3)	4025 (20.9)	4655 (21.7)	
Weekly alcohol use, g	74.9 (136.6)	115.0 (172.3)	38.9 (77.4)	<0.001
Alcohol use category				<0.001
Lowest	33,535 (86.3)	14,645 (79.6)	18,890 (92.3)	
Middle	3942 (10.1)	2688 (14.6)	1254 (6.1)	
Highest	1383 (3.6)	1064 (5.8)	319 (1.6)	

^a^Unemployed, student, homemaker.

### Associations between baseline variables

**Fig. 1:**
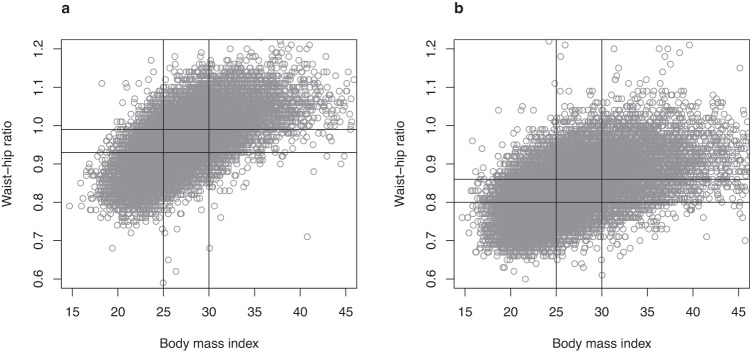
Correlations between waist-hip ratio and body mass index. Scatter plots showing the correlation between waist-hip ratio and body mass index in men (**a**) and women (**b**). Vertical lines represent the sex-specific tertile cutoffs. Horizontal lines separate normal weight from overweight and obesity.

WHR correlated with BMI among both men (*r* = 0.64, 95% CI 0.63–0.64, *P* < 0.001) and women (*r* = 0.54, 95% CI 0.53–0.55, *P* < 0.001) (Fig. 1). Nonetheless, 2128 (37%) of men and 4537 (53%) of women with a BMI 20–25 kg/m^2^ still had a WHR in the middle or highest sex-specific tertile. On the other hand, among men with a BMI above 30 kg/m^2^, 1211 (30%) had a WHR in the middle or lowest sex-specific tertile. Likewise, 1545 (33%) of women with BMI > 30 kg/m^2^ had a WHR in the middle or lowest tertile. The correlation between WHR and WC was 0.79 (95% CI 0.78–0.79, *P* < 0.001) for men and 0.81 (95% CI 0.80–0.81, *P* < 0.001) for women. Correlation coefficients between WHR and weekly alcohol use were 0.04 (*P* < 0.001) for men and −0.08 (*P* < 0.001) for women.

### Liver-related outcomes

We observed 355 liver-related outcome events during a median follow-up of 12.9 years (IQR 7.8–17.8, 509,047.8 person-years). Of the liver events, 198 (56%) were initially coded as alcohol-related liver disease, 109 (31%) as other chronic liver disease, and 48 (13%) as hepatocellular carcinoma. There were respectively 185, 83, and 71 liver-related events in the lowest, middle, and highest alcohol use strata. The respective figures for the WHR strata were 84, 98, and 173, and for the BMI strata, 87, 139, and 116. In addition, 11 liver events occurred in those with BMI < 20 kg/m^2^.

### WHR is a better predictor of liver-related outcomes than BMI

**Fig. 2:**
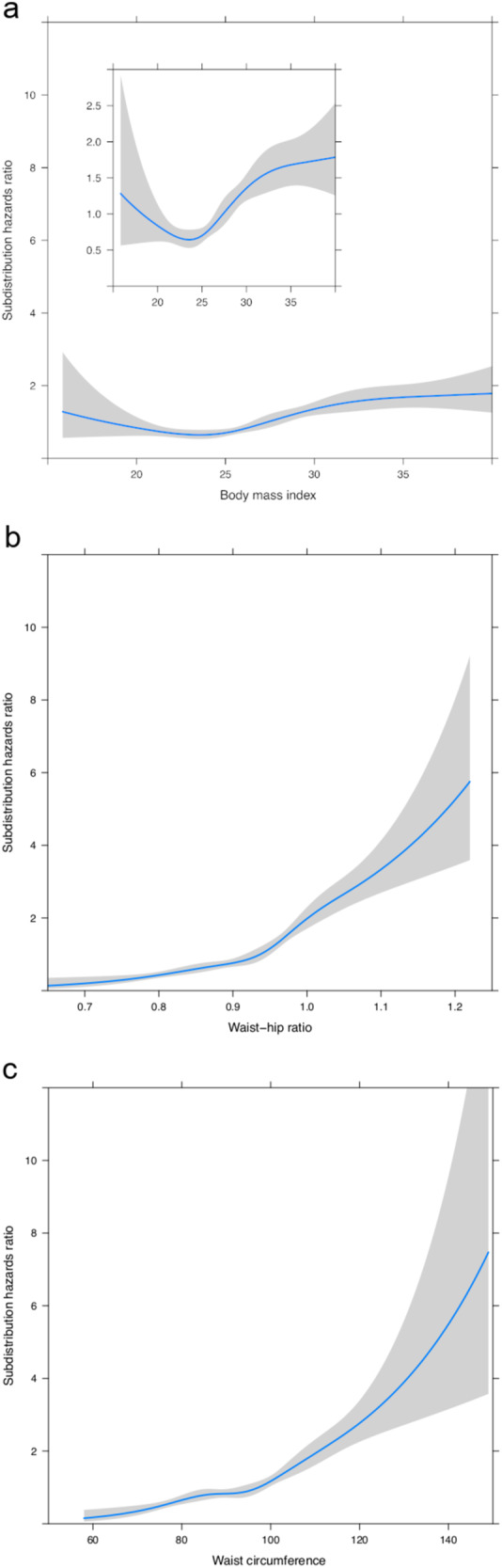
Anthropometric measures and liver-related outcomes. Influences on risk for liver-related outcomes of body mass index (**a**), waist-hip ratio (**b**), and waist circumference (**c**) by Fine-Gray regression analysis accounting for non-linear associations. Shaded areas represent 95% confidence intervals.

By univariate Fine-Gray regression analysis accounting for non-linear associations, there was no evidence that the association between WHR and the rate of liver-related outcomes was non-linear (*P* for non-linearity, 0.22); the same was true for WC and liver outcomes (*P* for non-linearity, 0.44) (Fig. 2). Regarding BMI, the association was *U*-shaped (*P* for non-linearity, 0.01), meaning that both a low and a high BMI increased the rate of liver-related outcomes, compared to a BMI around 20–25 kg/m^2^. In addition, WHR and WC emerged as more powerful predictors of liver-related outcomes than BMI, since the hazard ratios per 1 SD were 1.99 (95% CI 1.83–2.15) for WHR, 1.79 (95% CI 1.63–1.97) for WC, but only 1.35 (95% CI 1.23-1.48) for BMI. Even after the removal of those with BMI < 20 kg/m^2^ (i.e., the subgroup that caused the non-linear association between BMI and liver-related outcomes), the hazard ratio per 1 SD was 1.37 (95% CI 1.25–1.50) for BMI.

The discrimination performance (10-year AUC values) of WHR for liver-related outcomes was superior to that of BMI and WC, overall and separately among men and women, although the 95% CIs became wide in sex-specific analyses (Table 2).

**Table 2:** Area-under-the-curve (AUC) values at 10 years for the various anthropometric measures in predicting liver-related outcomes.

	All		Men		Women	
	10-year AUC (95% CI)	*P*^a^	10-year AUC (95% CI)	*P*^a^	10-year AUC (95% CI)	*P*^a^
Waist-hip ratio	0.714 (0.685–0.743)		0.697 (0.489–0.905)		0.679 (0.464–0.895)	
Waist circumference	0.648 (0.617–0.679)	<0.001	0.640 (0.445–0.835)	0.002	0.611 (0.414–0.808)	0.02
Body mass index (linear)	0.550 (0.514–0.585)	<0.001	0.568 (0.392–0.745)	<0.001	0.568 (0.380–0.757)	0.02
Body mass index (non-linear)	0.598 (0.557–0.640)	<0.001	0.619 (0.570–0.667)	0.006	0.573 (0.495–0.651)	0.02

Analyses are by Fine-Gray regression considering death without liver outcomes as a competing-risk event.

^a^Compared to the waist-hip ratio.

In the subgroup of safe and current alcohol users (*n* = 27,781 with 149 liver events), 10-year AUC values for WHR, BMI, and WC were 0.70 (95% CI 0.64–0.76), 0.61 (95% CI 0.54–0.68), and 0.67 (95% CI 0.60–0.73), respectively. In the subgroup of lifetime alcohol abstainers (*n* = 3587 with 16 liver events), the corresponding AUC values were 0.67 (95% CI 0.46–0.88), 0.64 (95% CI 0.40–0.88), and 0.68 (0.47–0.88), respectively.

### Effect modification and combined associations of abdominal obesity and BMI for liver-related outcomes

When examined in BMI strata, the superiority of WHR over WC in terms of discrimination was most clear in individuals with normal weight or overweight according to the BMI (Table 3).

**Table 3:** Area-under-the-curve (AUC) values at 10 years for waist-hip ratio and waist circumference in body mass index strata.

		10-year AUC		
Body mass index, kg/m^2^	*n*/*N*	Waist-hip ratio	Waist circumference	*P*^a^
<20	11/1433	0.871 (0.737–1.000)	0.812 (0.692–0.933)	0.20
20–24	87/14353	0.749 (0.567–0.931)	0.660 (0.494–0.826)	<0.001
25–29	139/16297	0.711 (0.504–0.918)	0.668 (0.473–0.864)	0.02
30–34	84/6364	0.730 (0.567–0.894)	0.723 (0.558–0.888)	0.70
≥35	32/2316	0.687 (0.539–0.835)	0.741 (0.597–0.886)	0.20

^a^Comparison between waist-hip ratio and waist circumference.

**Fig. 3:**
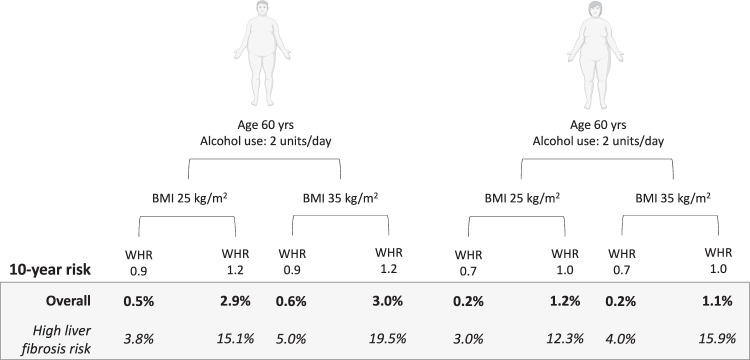
Estimated risks of liver-related outcomes according to the anthropometric profile. The effects of waist-hip ratio (WHR) and body mass index (BMI) on the absolute 10-year risk of liver-related outcomes (%) in men and women when age and alcohol use are kept constant. Risks are shown for the population overall and separately for those with high risk of having advanced liver fibrosis at baseline according to the dynamic aspartate aminotransferase-to-alanine aminotransferase ratio (dAAR) score. Analyses are by Fine-Gray competing-risk regression.

Figure 3 shows how the absolute risks of liver-related outcomes change, for example, individuals when age and alcohol use are kept constant, but WHR and BMI vary. As demonstrated, the absolute risks are more dependent on WHR than BMI. Furthermore, when the same analysis was repeated in the subgroup of 389 individuals with elevated baseline dAAR scores (i.e., high risk of having advanced liver fibrosis at baseline), the absolute risk estimates for liver-related outcomes increased substantially, highlighting how the effects of WHR becomes even more relevant when the background risk increases (Fig. 3).

### Supra-additive interaction between WHR and harmful alcohol use for liver-related outcomes

**Fig. 4:**
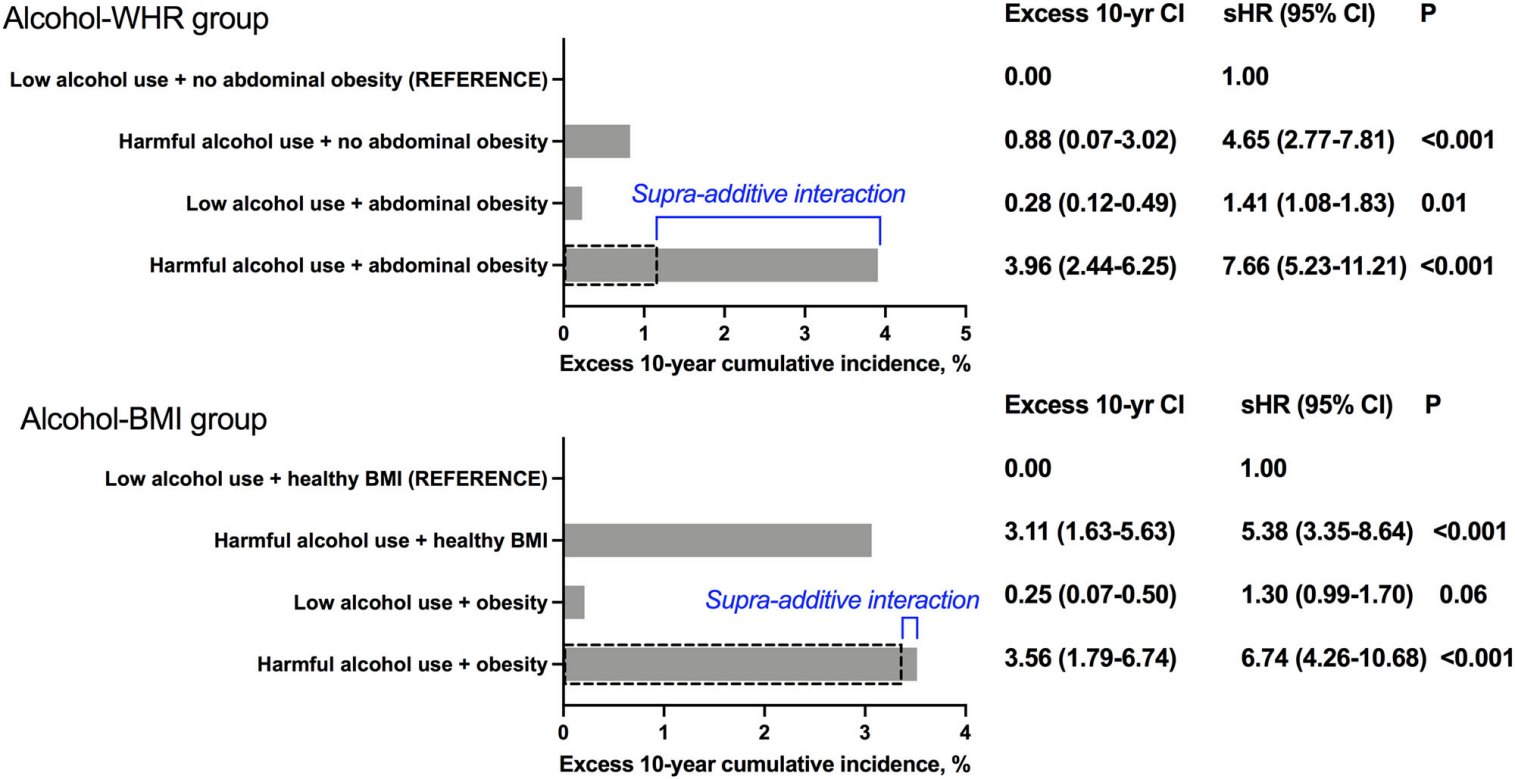
Interaction between harmful alcohol use and anthropometric measures. Excess cumulative incidence at 10 years of liver-related outcomes according to harmful alcohol use and waist-hip ratio (WHR) or body mass index (BMI).

Next, we assessed the excess cumulative incidence of severe liver disease after 10 years of follow-up according to harmful alcohol use and the highest risk category of WHR or BMI. When compared to the incidence of the reference group with low alcohol use and the lowest sex-specific WHR tertile (no abdominal obesity), those with harmful alcohol use and in the lowest WHR tertile (no abdominal obesity) had an excess cumulative incidence of liver-related outcomes of 0.88% at 10 years (Fig. 4). Those in the highest sex-specific WHR tertile (abdominal obesity) with low alcohol use had an excess incidence of 0.28%. Finally, among those in the highest sex-specific WHR tertile (abdominal obesity) and with harmful alcohol use, the excess cumulative incidence was 3.96% at 10 years, resulting in a supra-additive interaction effect equal to 2.80% from the combination of abdominal obesity and harmful alcohol use (Fig. 4). With regard to BMI, a similar interaction effect with harmful alcohol use was minimal (0.2%) and non-significant (Fig. 4). Similar supra-additive interaction effects between WHR and harmful alcohol use, but not between BMI and harmful alcohol use, were confirmed in multivariable-adjusted Fine-Gray regression analyses (Fig. 4).

When using the middle sex-specific WHR strata instead of the highest strata to define abdominal obesity, a supra-additive interaction effect equal to 2.77% at 10 years from the combination of abdominal obesity and harmful alcohol use remained. When considering the middle strata of BMI, the interaction effect was negative (−0.97% at 10 years).

## Discussion

We found that WHR predicted liver-related outcomes in the general population better than BMI or WC. Moreover, the performance of WHR was preserved in various BMI strata. We further demonstrated that the absolute risk of liver-related outcomes is substantially more dependent on the WHR than on the BMI, when age, sex, and alcohol use are kept constant. The superiority of WHR over the other anthropometric measures for predictions of liver-related outcomes has previously been demonstrated in two studies^6,7^. This evidence suggests that the WHR should become the standard obesity measure when assessing risk for liver-related outcomes. We acknowledge, however, that this might differ by ethnicity, which calls for further study in diverse ethnic groups. Nonetheless, any lack of WHR data in many datasets and clinical databases should not impede the introduction of the WHR in future studies and clinical practice.

Besides better predictive performance, WHR showed a significant supra-additive interaction effect with harmful alcohol use for liver-related outcomes, which was not seen between BMI and harmful alcohol use. This might explain the mixed findings regarding interaction effects between obesity and alcohol use in previous studies, most of which have assessed obesity using BMI^10–12^. WHR might better reflect metabolic health than BMI^2,3^. Metabolic dysfunction might sensitize the liver to the harmful effects of alcohol, as suggested by animal studies^20,21^ and human experimental studies^22^ and substantiated by recent epidemiologic evidence^23–25^.

The WHR is incorporated in the recent Chronic Liver Disease (CLivD) risk score, which can be used to predict incident liver-related outcomes in the general population^26^. The CLivD score simultaneously considers both alcohol use, abdominal obesity (WHR), age, sex, diabetes, smoking, and gamma-glutamyltransferase. The CLivD score has been validated in external cohorts^26,27^.

BMI showed a *U*-shaped association with liver-related outcomes, which has also been reported previously^28^. Based on this, it seems that losing weight is not necessarily always a good thing if this weight loss means that the individual is losing muscle mass, for example, due to illness. In contrast, losing weight in a way that reduces the WC in relation to the hip circumference emerges as a better marker of reduction of liver risk^29^.

It is noteworthy that, even at normal BMI levels (20–25 kg/m^2^), a substantial proportion of men and women had elevated WHRs. This calls to question the appropriateness of using BMI to define a lean type of NAFLD. The WHR might more accurately separate abdominally obese (metabolically unhealthy) NAFLD from non-abdominally obese NAFLD.

We found that body fat distribution reflected by the WHR was a stronger determinant of absolute risks for liver-related outcomes than BMI. Although the absolute risks remained low in our example individuals shown in Fig. 3 in the general population overall, we found that the absolute risks increased substantially in the subgroup of individuals with a high risk of having advanced liver fibrosis/cirrhosis at baseline. This supports the need for assessment of liver fibrosis as part of risk stratification strategies.

The WHR showed superior performance over WC in our study. Based on the same dataset, Danielsson et al. previously found that hip circumference has independent predictive value for liver-related outcomes^4^. However, the added predictive information from hip circumference is largely captured in the WHR, even though the WHR does not reflect the absolute values of WC or hip circumference, only their ratio^4^.

Strengths of our study include the large dataset representative of the general population and with long-term follow-up for clinical liver-related outcomes. To our knowledge, this is the first study to assess the predictive performance of different anthropometric measures for liver-related outcomes using competing-risk analysis.

In this study, WHR was measured using a standardized protocol. However, the validity of self-measurements has been confirmed previously^30,31^, and self-measurements could help make WHR more attainable in clinical practice. Recent mobile applications enable valid WHR measurements using digital photography technology^32^, and the introduction of such applications to the clinic is awaited to further increase the implementation of the WHR.

Study limitations include the relatively low number of liver-related outcome events and the reliance on registry-based outcomes. The Finnish population is predominantly white people, which might have an effect on results. Nonetheless, WHR has shown superiority over BMI also, for instance, in some previous Chinese studies^5,33^.

In conclusion, WHR predicted liver-related outcomes better than BMI or WC in our large cohort representative of the general population. We found supra-additive interaction effects between WHR and harmful alcohol use for liver-related outcomes, which were not seen between BMI and harmful alcohol use. In white people of European descent, the WHR merits to become the standard obesity measure when assessing risk for liver-related outcomes. Therefore, WHR needs to be implemented in clinical practice.

### Supplementary information

Supplementary Information

Reporting Summary

## Data Availability

FINRISK and Health 2000 data are available from the THL biobank based on a research application, as explained on the website of the THL biobank (https://thl.fi/en/web/thl-biobank/for-researchers). Source data for the figures contain individual-level data that cannot be made publicly available, but these data can be retrieved through research applications to the THL biobank.
