# Supplementary material for: Waist-hip ratio is superior to BMI in predicting liver-related outcomes and synergizes with harmful alcohol use
Source: Commun Med (Lond). 2023 Sep 6;3:119. doi: 10.1038/s43856-023-00353-2 (PMC10482890; doi:10.1038/s43856-023-00353-2)
Supplement: Supplementary file 1 — Supplementary Information [file 43856_2023_353_MOESM1_ESM.pdf]

Supplementary Note 1. ICD-codes used to define liver-related outcomes.

ICD-10 code	Diagnosis
K70.1	Alcoholic hepatitis
K70.2	Liver fibrosis caused by alcohol
K70.3	Alcoholic cirrhosis
K70.4	Liver failure related to alcohol
K70.9	Liver disease caused by alcohol
K72.0	Acute liver failure
K72.1	Chronic liver failure
K72.9	Liver failure unspecified
K74.0	Liver fibrosis
K74.1	Liver sclerosis
K74.2	Liver fibrosis and sclerosis
K74.6	Liver cirrhosis unspecified
K76.7	Hepatorenal syndrome
I85.0	Esophageal varices with bleeding
I85.9	Esophageal varices without bleeding
C22.0	Hepatocellular carcinoma
**ICD-9 code**	
571.1	Acute alcoholic hepatitis
571.2	Alcoholic cirrhosis of liver
571.3	Alcoholic liver damage, unspecified

571.5	Cirrhosis of liver without mention of alcohol
571.8	Other chronic nonalcoholic liver disease
572.2	Hepatic encephalopathy
572.4	Hepatorenal syndrome
572.8	Other sequelae of chronic liver disease
456.0	Esophageal varices with bleeding
456.1	Esophageal varices without mention of bleeding
155.0	Liver cancer
**ICD-8 code**	
571.0	Alcoholic cirrhosis
571.8	Cirrhosis, other
571.9	Cirrhosis, unspecified
573.0	Hepatitis NUD
573.9	Other liver disease
155.01	Liver cancer
