# Supplementary material for: Waist-hip ratio is superior to BMI in predicting liver-related outcomes and synergizes with harmful alcohol use
Source: Commun Med (Lond). 2023 Sep 6;3:119. doi: 10.1038/s43856-023-00353-2 (PMC10482890; doi:10.1038/s43856-023-00353-2)
Supplement: Supplementary file 2 — Reporting Summary [file 43856_2023_353_MOESM2_ESM.pdf]

nature portfolio

Corresponding author(s): Fredrik Åberg

Last updated by author(s): Aug 9, 2023

Reporting Summary

Nature Portfolio wishes to improve the reproducibility of the work that we publish. This form provides structure for consistency and transparency in reporting. For further information on Nature Portfolio policies, see our Editorial Policies and the Editorial Policy Checklist.

Statistics

For all statistical analyses, confirm that the following items are present in the figure legend, table legend, main text, or Methods section.

n/a	Confirmed	
☐	☒	The exact sample size (*n*) for each experimental group/condition, given as a discrete number and unit of measurement
☐	☒	A statement on whether measurements were taken from distinct samples or whether the same sample was measured repeatedly
☐	☒	The statistical test(s) used AND whether they are one- or two-sided<br>*Only common tests should be described solely by name; describe more complex techniques in the Methods section.*
☐	☒	A description of all covariates tested
☐	☒	A description of any assumptions or corrections, such as tests of normality and adjustment for multiple comparisons
☐	☒	A full description of the statistical parameters including central tendency (e.g. means) or other basic estimates (e.g. regression coefficient) AND variation (e.g. standard deviation) or associated estimates of uncertainty (e.g. confidence intervals)
☐	☒	For null hypothesis testing, the test statistic (e.g. *F*, *t*, *r*) with confidence intervals, effect sizes, degrees of freedom and *P* value noted<br>*Give P values as exact values whenever suitable.*
☒	☐	For Bayesian analysis, information on the choice of priors and Markov chain Monte Carlo settings
☒	☐	For hierarchical and complex designs, identification of the appropriate level for tests and full reporting of outcomes
☐	☒	Estimates of effect sizes (e.g. Cohen's *d*, Pearson's *r*), indicating how they were calculated

Our web collection on statistics for biologists contains articles on many of the points above.

Software and code

Policy information about availability of computer code

Data collection	Microsoft Excel, R software version 3.6.1
Data analysis	R software version 3.6.1.

For manuscripts utilizing custom algorithms or software that are central to the research but not yet described in published literature, software must be made available to editors and reviewers. We strongly encourage code deposition in a community repository (e.g. GitHub). See the Nature Portfolio guidelines for submitting code & software for further information.

Data

Policy information about availability of data

All manuscripts must include a data availability statement. This statement should provide the following information, where applicable:

- Accession codes, unique identifiers, or web links for publicly available datasets
- A description of any restrictions on data availability
- For clinical datasets or third party data, please ensure that the statement adheres to our policy

FINRISK and Health 2000 data are available from the THL biobank based on a research application, as explained on the website of the THL biobank (https://thl.fi/en/web/thl-biobank/for-researchers). Source data for the figures contain individual level data that cannot be made publicly available, but these data can be retrieved through research application to the THL biobank.

Research involving human participants, their data, or biological material

Policy information about studies with human participants or human data. See also policy information about sex, gender (identity/presentation), and sexual orientation and race, ethnicity and racism.

Reporting on sex and gender	Sex is based on self-reports. We report sex-specific results.
Reporting on race, ethnicity, or other socially relevant groupings	We report marital status, employment status and education level based on questionnaire data and prespecified categories (Table 1). We do not report data on race/ethnicity.
Population characteristics	See above
Recruitment	Randomly selected adults from the general population were invited to participate in the health-examination surveys, please refer to the methods section of the manuscript for more information
Ethics oversight	The Coordinating Ethical Committee of the Helsinki and Uusimaa Hospital District. Previously, the studies were also approved by the institutional review board of the National Public Health Institute in Helsinki, Finland.

Note that full information on the approval of the study protocol must also be provided in the manuscript.

Field-specific reporting

Please select the one below that is the best fit for your research. If you are not sure, read the appropriate sections before making your selection.

☒ Life sciences ☐ Behavioural & social sciences ☐ Ecological, evolutionary & environmental sciences

For a reference copy of the document with all sections, see nature.com/documents/nr-reporting-summary-flat.pdf

Life sciences study design

All studies must disclose on these points even when the disclosure is negative.

Sample size	Each sample (FINRISK 1992, 1997, 2002, 2007, 2012, Health 2000) determined their sample size as previously reported to achieve good representation of the general population in Finland. We included here all subjects that participated and had sufficient data available.
Data exclusions	We excluded individuals with missing registry linkage (n=1457), baseline liver disease (ICD-10 codes K70-K77 or C22; n=299), chronic viral hepatitis (n=89), or missing anthropometric measurements (n=338).
Replication	Inclusion of 6 different samples (FINRISK 1992, 1997, 2002, 2007, 2012, Health 2000) increases replicability of the findings.
Randomization	Not a randomized study
Blinding	Grouping and exposure variables were specific to this study, so study subjects and investigators were unaware of these characteristics at the time of data collection.

Reporting for specific materials, systems and methods

We require information from authors about some types of materials, experimental systems and methods used in many studies. Here, indicate whether each material, system or method listed is relevant to your study. If you are not sure if a list item applies to your research, read the appropriate section before selecting a response.

Materials & experimental systems

n/a	Involved in the study
☒	☐ Antibodies
☒	☐ Eukaryotic cell lines
☒	☐ Palaeontology and archaeology
☒	☐ Animals and other organisms
☒	☐ Clinical data
☒	☐ Dual use research of concern
☒	☐ Plants

Methods

n/a	Involved in the study
☒	☐ ChIP-seq
☒	☐ Flow cytometry
☒	☐ MRI-based neuroimaging
